# Patient preferences for treatment of castration-resistant prostate cancer in Japan: a discrete-choice experiment

**DOI:** 10.1186/s12894-016-0182-2

**Published:** 2016-11-04

**Authors:** Hiroji Uemura, Nobuaki Matsubara, Go Kimura, Akito Yamaguchi, Dianne Athene Ledesma, Marco DiBonaventura, Ateesha F. Mohamed, Enrique Basurto, Ian McKinnon, Ed Wang, Kristen Concialdi, Aya Narimatsu, Yasuko Aitoku

**Affiliations:** 1 Department of Urology and Renal Transplantation, Yokohama City University Medical Center, Yokohama, Japan; 2 Division of Breast and Medical Oncology, National Cancer Center Hospital East, Chiba, Japan; 3 Department of Urology, Nippon Medical School, Tokyo, Japan; 4 Division of Urology, Harasanshin General Hospital, Fukuoka, Japan; 5 Bayer Yakuhin, Ltd., 2-4-9, Umeda, Kita-ku, Osaka 530-0001 Japan; 6 Kantar Health, New York, NY USA; 7 Bayer Healthcare, Whippany, NJ USA

**Keywords:** Castration-resistant prostate cancer, Patient preferences, Fatigue, Bone pain, Symptomatic

## Abstract

**Background:**

Up to a fifth of patients diagnosed with prostate cancer (PC) will develop castration-resistant prostate cancer (CRPC), which has been associated with a poor prognosis. The aim of this study was to consider the patient perspective as part of the overall treatment decision-making process for CRPC, given that an alignment between patient preference and prescribing has been shown to benefit patient outcomes. This study examines preferences of patients with CRPC in Japan for treatment features associated with treatments like RA-223, abiraterone, and docetaxel and to examine the extent to which treatment preferences may vary between symptomatic and asymptomatic patients.

**Methods:**

A two-phase research approach was implemented. In Phase 1, *N* = 8 patients with CRPC were recruited from a single hospital to complete a qualitative interview to provide feedback on the draft survey. In Phase 2, *N* = 134 patients with CRPC were recruited from five hospitals to complete a paper survey. The survey included 6 treatment choice questions, each asking patients to choose between two hypothetical treatments for their CRPC. Each treatment alternative was defined by the following attributes: length of overall survival (OS), time to a symptomatic skeletal event (SSE), method of administration, reduction in the risk of bone pain, treatment-associated risk of fatigue and lost work days. A hierarchical Bayesian logistic regression was used to estimate relative preference weights for each attribute level and mean relative importance.

**Results:**

A total of *N* = 133 patients with CRPC completed the survey and were included in the final analysis. Patients had a mean age of 75.4 years (SD = 7.4) and had been diagnosed with PC a mean of 6.5 years prior (SD = 4.4). Over the attribute levels shown, fatigue (relative importance [RI] = 24.9 %, 95 % CI: 24.7 %, 25.1 %) was the most important attribute, followed by reduction in the risk of bone pain (RI = 23.2 %, 95 % CI: 23.0 %, 23.5 %), and OS (RI = 19.2 %, 95 % CI: 19.0 %, 19.4 %). Although symptomatic patients placed significantly more importance on delaying an SSE (*p* < .05), no other preference differences were observed.

**Conclusions:**

CRPC patients were more concerned about reduced quality of life from side effects of treatment rather than extension of survival, which may have implications for shared decision-making between patients and physicians.

## Background

Prostate cancer (PC) is the fourth-most common cancer in Japan, with most patients being diagnosed at 70 years or older [1]. Since the middle of 2014, there have been a number of advancements in the treatment options for PC, specifically in metastatic castration-resistant PC (mCRPC) (e.g., abiraterone, enzalutamide, cabazitaxel, Ra-223) in Japan, which has extended overall survival (OS) significantly based on available clinical data [2–7]. Yet, these treatment options can vary substantially with respect to their effectiveness, safety profile and method of administration, among other characteristics [2], complicating treatment decision-making for patients and physicians.

In an extensive review of patient preferences and decision-making aids in the context of PC, Aning and colleagues reiterated the importance of medical care that seeks to adopt shared decision-making and aligns treatment decisions with patient goals and values [8]. As the complexity of cancer treatment increases globally, the role of patient preferences in guiding medical care will become increasingly critical [9–13].

Research has suggested that patient preferences, in the context of cancer, possess implications for adherence, persistence and follow-up care [14]. If patient preferences and prescribed treatment regimens are misaligned, patients can be at an increased risk for discontinuation and non-adherence, which could affect symptom management and survival [14]. As a result, it is important to understand how patients value different treatments to best inform overall disease management.

Studies in the United States and Europe have been conducted to explore patient preferences among patients with PC, with a focus on the treatment of bone metastases [15–17]. The results from these studies suggest that patients place substantial value on the delay of bone complications; indeed, in one study, patients were willing to sacrifice 3–5 months of survival in the interest of avoiding them [15].

Critically, however, no study has examined how patients in Japan value differences in the characteristics of CRPC treatment options and how these preferences differ based on the symptomatic status of the patient. As research suggests significant variation in patient preferences across countries and cultures, there is a need for more Japan-specific studies [18]. Therefore, the objective of this study was to fill this important gap in the literature by enhancing the understanding of treatment preferences among patients with CRPC in Japan.

## Methods

### Sample and procedure

A discrete-choice experiment (DCE) method was used. The DCE is a survey approach designed to assess respondents’ willingness to accept tradeoffs among hypothetical treatment profiles described by treatment attributes of varying levels. In a healthcare context, the attractiveness of a treatment to patients depends on patients’ relative preferences for treatment attributes expressed by their willingness to accept tradeoffs among them.

This study was designed to follow the good practice guidelines for conjoint analysis associated with the International Society of Pharmacoeconomics and Outcomes Research [19]. A two-phase approach was conducted. In the first phase (Phase 1), a draft paper-and-pencil survey was piloted on a small group of Japanese patients with CRPC (*N* = 8) in a qualitative research setting to solicit feedback and to determine the content validity of the survey (i.e., ensuring the DCE materials were appropriate and sufficiently clear to patients). Specifically, a participating physician from one hospital in Japan identified patients with CRPC who met eligibility criteria and were willing to participate. Inclusion criteria were as follows: (1) aged 20 years or older, (2) diagnosed with CRPC, (3) chemotherapy-naïve, (4) able to read and understand Japanese, and (5) provided written informed consent. Patients were excluded if they were currently participating in a clinical trial, unable to complete the survey by themselves due to physical or psychological reasons, or were otherwise deemed ineligible by the referring physician.

In the second phase (Phase 2), the paper-and-pencil survey was distributed to *N* = 134 pre-selected CRPC patients at four participating sites, who were identified by various hospital-based clinicians/investigators. The inclusion and exclusion criteria, as well as the sequencing of the items in the questionnaire, were fixed across respondents and were the same as those used in Phase 1. Patients completed the survey in their home and called a researcher to provide their responses, which were then entered into an electronic database. The researcher ensured that patients’ responses were recorded for all components of the questionnaire. Basic clinical measures (as described below), provided by the patients’ respective physicians, were also entered into this same database and integrated into the analyses.

### Design efficiency

The experimental design in a choice-exercise survey involves a careful selection, achieving statistical properties of balance and orthogonality, of possible combinations among the full set of theoretical combinations of attributes and levels. An orthogonal design implies attributes and levels tested independently from one another, ensuring that importance contributions of individual features can be isolated from the remaining (possibly confounding) effects presented simultaneously as part of the DCE. A balanced design implies that all attributes and levels are being exposed an equal number of times in the DCE. Equal exposure implies that each level has an equal chance of being selected.

Given the expected number of possible combinations and the information necessary to complete each task, a full profile, fractional, factorial, balanced, incomplete block design was used to maximize the number of exposures of the attribute levels in each choice task and across the experimental design. D-efficiency values range from 0 to 100, with higher values reflecting a more balanced and orthogonal design, with minimized variability of estimates obtained through the DCE and consequently more accurate results for main effects and interactions [20]. Assuming one 5-level and five 3-level attributes (with the expected achievable sample size of 134 noted above), the number of possible combinations was computed at 1,476,225.

The D-efficiency is determined by the number of cards included and the ability to achieve balance and orthogonality. Warren Kuhfeld’s macro *%MktRuns* was used to calculate reasonable design sizes [20]. The selection of the total number of cards was based on maximizing the accuracy of the model results: namely, choosing the most cards possible to maximize observed design points for analysis, while also choosing the fewest cards possible per respondent to minimize fatigue and the fewest choice sets possible that are not divisible by any 2-way combination of the attributes and levels considered (i.e., “violations”). Based on these selection criteria, a design containing 45 cards in 9 blocks was chosen, keeping the number of cards per respondent and the number of violations to a minimum.

An additional holdout card was then inserted in all blocks containing the head-to-head comparison between the absolute best case scenario (i.e., the hypothetical profile containing all the most desirable features of the treatment) and the absolute worst case scenario (i.e., the hypothetical profile containing the least desirable features of the treatment). This card was inserted in order to determine the respondents’ ability to identify the profile with dominant characteristics over the one with the least desirable characteristics. Respondents who did not choose the profile with clearly dominant characteristics were flagged and then reported as a “risk ratio” (see Validity Assessments section). Hence, the total number of cards seen by a given respondent was six, for a final (augmented) design containing 54 cards in 9 blocks.

The appropriate selection of the design size is key to maximize the efficiency of the design. Kuhfeld’s macro * %MktEx* was then used to create the combinations that could maximize the efficiency of the design for a resulting D-efficiency of 90.51 [20].

### Measures

#### Choice task

As previously noted, the survey itself included six preference-elicitation questions, each asking respondents to choose between two hypothetical treatments for their PC (no real treatments or treatment names were used or mentioned in the survey; instead they were presented as “Medicine A” and “Medicine B”). Each hypothetical treatment alternative shown in the preference-elicitation questions was defined by six attributes, which were identified through literature search and expert medical opinion (urologists who treat mCRPC patients). The specific attributes were as follows: (1) method of administration, (2) OS, (3) time to a symptomatic skeletal event (SSE), (4) reduction in the risk of bone pain, (5) risk of fatigue and (6) lost work days following treatment. The specific levels of each attribute, which were developed by extracting data from the clinical studies provided in the labels for RA-223, abiraterone and docetaxel, are shown in Table 1.

**Table 1:** Attributes and levels represented in the DCE

Attribute	Levels
Administration	Six 1-min IV injections (requiring 5 h in the hospital) every 4 weeks; no radiation emitted
	Six 1-min IV injections (requiring 5 h in the hospital) every 4 weeks; minor radiation emitted
	Six 1-min IV injections (requiring 5 h in the hospital) every 4 weeks; some radiation emitted
	Four pills taken orally once a day; 1 h at the hospital every 2 weeks
	Six 1-h IV infusions (requiring 1–2 weeks in the hospital the first time and 7–8 h each other time) every 3 weeks
OS	14 months
	16 months
	20 months
Time to SSE	10 months
	14 months
	16 months
Reduction in the risk of bone pain	25 % chance of suppressing bone pain
	50 % chance of suppressing bone pain
	75 % chance of suppressing bone pain
Risk of fatigue	0 % chance of fatigue
	30 % chance of fatigue
	60 % chance of fatigue
Work loss due to treatment	0 days
	3 days
	5 days

**Fig. 1:**
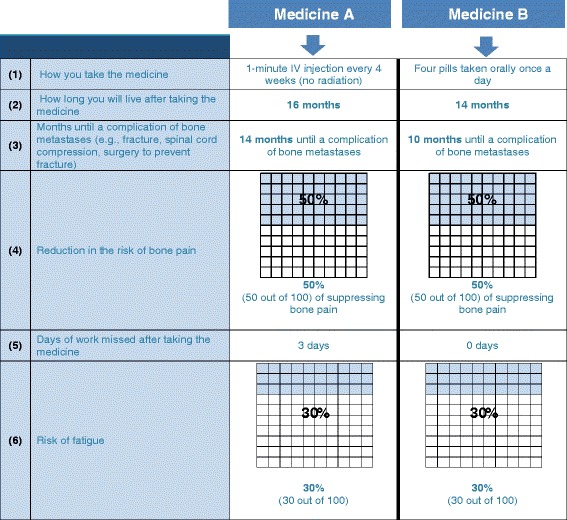
Example preference elicitation task

The experimental design ensured a sufficient number of patients saw the different combinations of attributes and levels. All attribute levels varied independently according to this experimental design, which dictated the number and types of choice questions presented to each respondent when assigned to a specific block. Figure 1 represents an example of a single preference-elicitation question that was presented to respondents. After reviewing the information in the two presented profiles, the patient was asked to select which of the two profiles (Medicine A or B) he would prefer as his PC treatment.

### Validity assessments

As previously discussed, a holdout card containing the head-to-head comparison between the absolute best and worst case scenarios was inserted in all blocks in order to determine the respondents’ ability to choose the profile with dominant characteristics over the one with the least desirable characteristics. Respondents who did not choose the profile options with dominant characteristics were flagged and reported as a “risk ratio.” The within-set risk ratio, referring to the percentage of respondents who selected the worst possible profile over the best one, was found to be 1.5 % (i.e., two respondents). Moreover, respondents were presented with other holdout cards in which one of the profiles had dominant characteristics on all attributes. For the across-set monotonicity risk ratio, the percentage of respondents making at least one incorrect selection in these other holdout cards was found to be 4.5 % (i.e., six respondents). The overall root likelihood was equal to 852, which meant that with six alternatives in each preference-elicitation question, the model was 5.1 times better at predicting the preference that respondents would have had for the alternatives presented, compared with chance. Goodness of fit of these models was also reflected in the observed 95 % confidence intervals ranging from +/−1 to 5 %, where the lowest margins were found at the extremes and the highest margins around the middle of the preference continuum. Based on these analyses, one patient was excluded due to inconsistent responding, yielding a final sample of 133 patients included in the main analyses.

#### Patient demographics and disease history

Physicians reported patient characteristics, including years since diagnosis, symptomatic status (“symptomatic” being defined based on regular analgesic/opioid use or external beam radiation therapy for bone pain), presence and number of metastases, Eastern Cooperative Oncology Group (ECOG) performance status, and history of an SSE. Patients self-reported their demographics (age, region of residence, education, household income, employment status, insurance status, etc.), comorbidities (which were used to calculate a Charlson comorbidity index [CCI] score for each patient [21, 22], height and weight (which were used to derive a body mass index [BMI] category), and level of pain in the past 24 h and in the past week.

### Statistical analysis

#### Descriptive statistics

The study sample was described with respect to demographics, disease history and comorbidity variables using frequencies and percentages for categorical variables and counts, means and standard deviations (SDs) for continuous variables.

#### Patient preferences

Data from the DCE were analyzed using a hierarchical Bayesian logistic regression model. The outcome variable of this model was choice (“Medicine A” vs. “Medicine B”) and the predictor variables were the levels within each attribute (i.e., method of administration, OS, time to an SSE, reduction in the risk of bone pain, risk of fatigue and lost work days following treatment). Effects coding was used for each level within each attribute. The resulting parameter estimate for each attribute level represents the preference weight, which is defined as the marginal utility of a change in that attribute. With the exception of method of administration, interpolations were made to determine the marginal utilities for the values contained within the numeric range tested for each attribute. Parameter estimates for the levels tested were reported, along with their standard errors, 95 % confidence intervals (CIs), and statistical significance.

These parameter estimates were also used to calculate relative importance weights using the MSS approach [23]. Attributes with higher relative importance weights hold stronger relationships with treatment choice than other attributes, and these weights are on a ratio scale (e.g., an attribute with a relative importance of 50 % is twice as important as an attribute with a relative importance of 25 %). Ninety-five percent CIs for these estimates were also reported.

## Results

### Sample characteristics

A total of *N* = 134 patients with CRPC completed the survey. One respondent (*N* = 1) was excluded from the analysis, as his responses to the DCE suggested he may not have fully understood the task, based on the validity assessments described above. The remaining *N* = 133 respondents had a mean age of 75.4 years (SD = 7.4) (Table 2), and most were retired (74.4 %), married (82.7 %), and from the Kanto region (83.5 %).

**Table 2:** Demographic characteristics of the study sample (*N* = 133)

	Total
	(*N* = 133)
Age (Mean ± SD)	75.36 ± 7.39
Four-year university (%)	49 (36.84 %)
Married (%)	110 (82.71 %)
Region	
Kanto (%)	111 (83.46 %)
Chubu (%)	1 (0.75 %)
Chugoku (%)	1 (0.75 %)
Kyushu (%)	20 (15.04 %)
Employed (%)	43 (32.33 %)
Household income	
Less than ¥2,500,000 (%)	34 (25.56 %)
¥2,500,000 to ¥4,999,999 (%)	57 (42.86 %)
¥5,000,000 to ¥7,499,999 (%)	19 (14.29 %)
¥7,500,000 or more (%)	15 (11.28 %)
Decline to answer (%)	8 (6.02 %)
Medical insurance	
National Health Insurance (%)	51 (38.35 %)
Late Stage Elderly Insurance (%)	66 (49.62 %)
Other (Company/Social Insurance) (%)	14 (10.53 %)
None of the above (all treatment costs paid by patient) (%)	2 (1.50 %)
Body mass index (BMI) category	
Underweight (<18.5 kg/m^2^) (%)	6 (4.51 %)
Acceptable risk (≥18.5 kg/m^2^ to <23 kg/m^2^) (%)	43 (32.33 %)
Increased risk (≥23 kg/m^2^ to <27.5 kg/m^2^) (%)	68 (51.13 %)
High risk (≥27.5 kg/m^2^) (%)	16 (12.03 %)
Charlson comorbidity index (CCI) (Mean ± SD)	5.16 ± 3.19

Patients had been diagnosed with PC a mean of 6.5 years prior (SD = 4.4) (Table 3). Over two-thirds (69.9 %) had metastatic disease, and over a quarter of these patients (25.8 %) had visceral metastases. A total of 45.1 % of patients had at least one bone metastasis, with 15.0 % of patients having 4 or more metastases. Over a fifth (20.3 %) of patients were symptomatic. Patients self-reported a high comorbidity burden (mean CCI = 5.2, SD = 3.2). Nearly 25 % of the patients reported experiencing pain in the past day and pain in the past week.

**Table 3:** Prostate cancer history of the study sample (*N* = 133)

	Total (*N* = 133)
Years diagnosed with PC	
Mean ± SD	6.45 ± 4.35
Metastatic disease (%)	93 (69.92 %)
Visceral metastases (of those with metastatic disease) (%)	24 (25.81 %)
Number of bone metastases	
None (%)	73 (54.89 %)
1 (%)	17 (12.78 %)
2 (%)	12 (9.02 %)
3 (%)	11 (8.27 %)
4+ (%)	20 (15.04 %)
Skeletal-related events	
Bone pain (%)	34 (25.56 %)
Bone fracture (%)	3 (2.26 %)
Spinal cord compression (%)	10 (7.52 %)
Bone surgery (%)	3 (2.26 %)
Radiation to the bone (%)	12 (9.02 %)
Other (%)	1 (0.75 %)
None (%)	93 (69.92 %)
Symptomatic status	
Symptomatic (%)	27 (20.30 %)
Asymptomatic (%)	106 (79.70 %)
ECOG performance status	
Grade 0 (%)	85 (63.91 %)
Grade 1 (%)	42 (31.58 %)
Grade 2 (%)	5 (3.76 %)
Grade 3 (%)	1 (0.75 %)

### Patient preferences

**Fig. 2:**
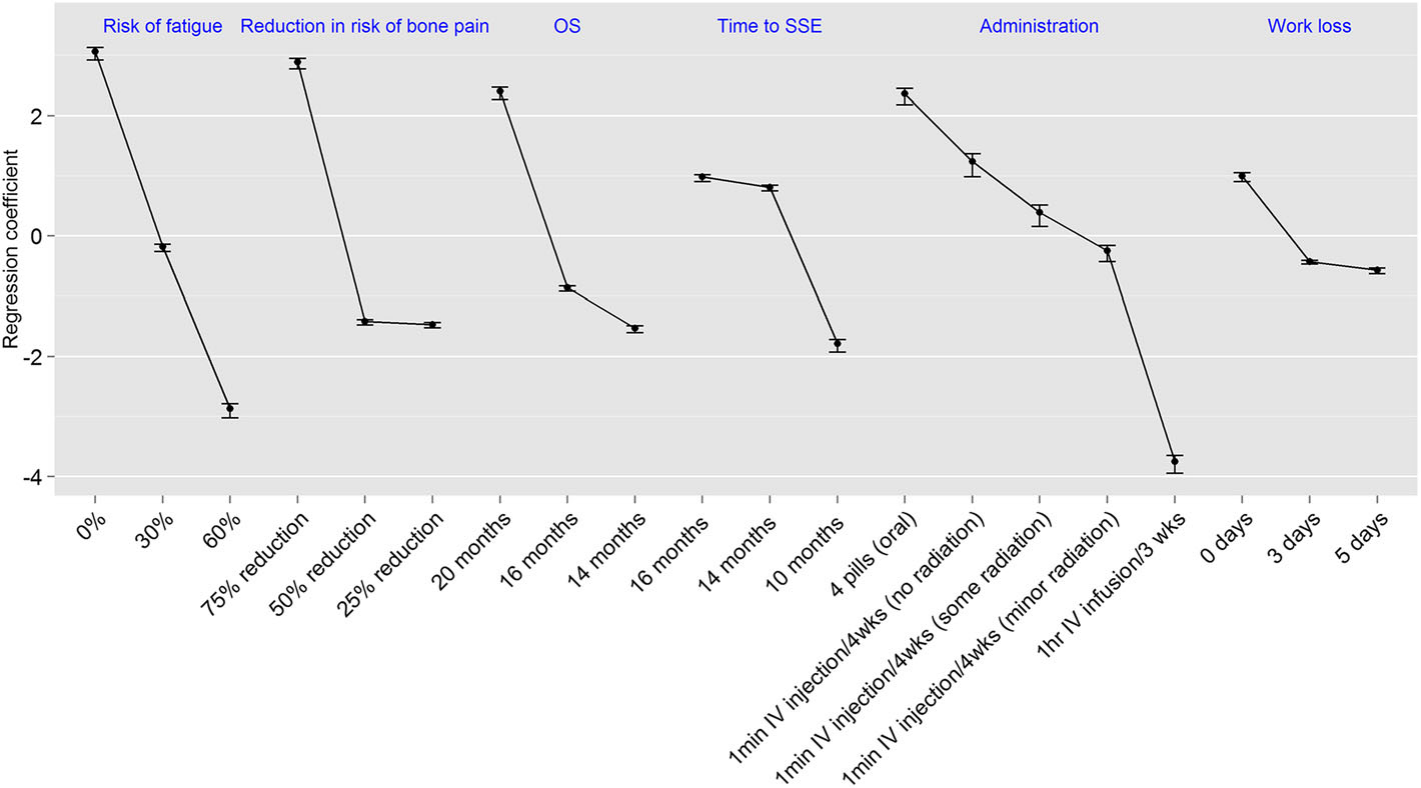
Patient preference weights (*N* = 133). Footnote: Bars represent 95 % confidence intervals. “Minor radiation” was described as radiation that can be stopped by a thin sheet of paper without any risk of contaminating others with the patient’s bodily fluids; “some radiation” was described as radiation which can be stopped by aluminum or lead and care must be taken not to contaminate others with bodily fluids for one week

**Fig. 3:**
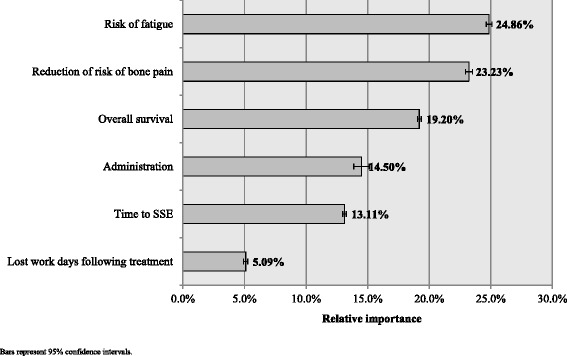
Importance of treatment attributes for patients (*N* = 133). Footnote: Bars represent 95 % confidence intervals

The full hierarchical Bayesian logistic regression model results are reported in Table 4. All levels of all attributes were significantly associated with choice (all *p* < .05). The preference weights and their 95 % confidence intervals are also displayed in Fig. 2. The greater the vertical changes within an attribute (as illustrated for risk of fatigue, reduction in risk of bone pain, and OS), the stronger is the relationship between that attribute and treatment choice. This is further illustrated in the relative importance weights in Fig. 3. Over the range of attributes and levels included in the survey, risk of fatigue (relative importance [RI] = 24.9 %, 95 % CI: 24.7 %, 25.1 %) was the most important attribute, followed by reduction in the risk of bone pain (RI = 23.2 %, 95 % CI: 23.0 %, 23.5 %), and OS (RI = 19.2 %, 95 % CI: 19.0 %, 19.4 %). Both risk of fatigue and reduction in the risk of bone pain were at least 50 % more important to choice than method of administration (RI = 14.5 %), time to an SSE (RI = 13.1 %), and lost work days following treatment (RI = 5.1 %).

**Table 4:** Regression model results predicting medication choice (*N* = 133)

Attribute	Levels	b	SE	Z	*p*-value	OR
Administration	1-min IV injection every 4 weeks (no radiation)	1.24	0.13	9.43	0.000	3.46
	1-min IV injection every 4 weeks (some radiation)	0.39	0.12	3.27	0.001	1.48
	1-min IV injection every 4 weeks (minor radiation)	−0.25	0.09	−2.82	0.005	0.78
	Four pills taking orally once a day	2.36	0.09	25.41	0.000	10.64
	1-h IV infusion every 3 weeks	−3.75	0.10	−38.27	0.000	0.02
Overall survival	14 months	−1.53	0.04	−36.80	0.000	0.22
	16 months	−0.86	0.03	−26.46	0.000	0.42
	20 months	2.40	0.07	33.87	0.000	11.00
Time to SSE	10 months until an SSE	−1.79	0.07	−26.14	0.000	0.17
	14 months until an SSE	0.81	0.03	24.56	0.000	2.25
	16 months until an SSE	0.98	0.04	27.38	0.000	2.67
Reduction in risk of bone pain	75 % chance of suppressing bone pain	2.89	0.06	50.66	0.000	17.93
	50 % chance of suppressing bone pain	−1.42	0.03	−49.53	0.000	0.24
	25 % chance of suppressing bone pain	−1.47	0.03	−51.73	0.000	0.23
Fatigue	0 % chance of fatigue	3.06	0.07	43.25	0.000	21.25
	30 % chance of fatigue	−0.18	0.04	−4.22	0.000	0.83
	60 % chance of fatigue	−2.87	0.08	−38.02	0.000	0.06
Lost work days following treatment	0 days	1.00	0.05	21.92	0.000	2.71
	3 days	−0.43	0.02	−20.63	0.000	0.65
	5 days	−0.57	0.03	−22.54	0.000	0.57

Odds ratios (ORs) represent the adjusted relative odds of selecting a specific level vs. the mean level within any given attribute

Upon examining specific levels of the attributes (Table 4 and Fig. 2), the results suggest that the risk of fatigue held a linear relationship with patient preference, with each increasing 1 % of fatigue having a uniform effect on treatment choice. With respect to method of administration, oral pills were strongly preferred over any IV injection and infusion; IV injection was strongly preferred over infusion (all *p* < .05). Also, an IV injection with no radiation was preferred over an IV injection with some radiation which, in turn, was preferred over an IV injection with minor radiation (all *p* < .05). The relationship between OS and preference was non-linear: a patient values a 4-month OS extension (16-20 months) higher than a 2-month OS extension (14–16 months). The relationship between time to an SSE and preference was also non-linear, such that an additional 1-month delay in an SSE was more important when the overall delay was 10–14 months, compared with an additional 1-month delay when the overall delay was 14–16 months (all *p* < .05).

The reduction in the risk of bone pain was also non-linear, such that each increasing 1 % of risk reduction was far less important to patients when the overall risk reduction was between 25 and 50 % than when the overall risk reduction was between 50 and 75 %. Indeed, the differences between 25 and 50 % in risk reduction were quite trivial and had little effect on treatment choice. Finally, lost work days also exhibited a similar pattern to reduction in the risk of bone pain. Although patients had a strong preference for 0 days lost, the difference in days lost between 3 and 5 days was relatively negligible.

### Patient preferences by symptomatic status

A final analysis compared differences in preference weights between patients who were symptomatic versus asymptomatic. With the exception of time to an SSE, no differences were observed. Symptomatic patients placed significantly more importance on delaying an SSE, as shown by a greater preference weight for 16 months to an SSE (b = 1.1 vs. 0.9, *p* < .05) and a lower preference weight for 10 months to an SSE (b = −2.1 vs. -1.7, *p* < .05).

### Patient preferences by demographic and health history

Additional comparisons of preference weights were made across demographic and health history variables. Given the lack of a priori hypotheses for these variables, a Bonferroni correction (ranging from α = 0.0083 for comparisons across four-category measures to an unadjusted α = 0.05 for comparisons across two-category measures) was made to minimize the possibility of a spurious finding. No significant differences in preferences weights were observed.

## Discussion

The findings suggest that patients with CRPC in Japan primarily value the risk of fatigue and reduction in the risk of bone pain when considering potential treatment options for their PC. Indeed, these characteristics were even more important (approximately 20 % to 30 % more important) than OS across the attributes and levels used in this study. Interestingly, although the risk of fatigue had a relatively linear relationship with preference, reduction in the risk of bone pain did not. Specifically, the benefit of a 30 % reduction was seen as relatively comparable to a 0 % reduction. A 60 % reduction, however, was considerably more valued by patients. Although this suggests a floor effort, in that only substantial risk reductions are valued by patients, it is unclear why this may be the case. Additional research is necessary.

The emphasis on minimizing risks, particularly symptomatic risks, over survival somewhat diverges from past research. Although symptomatic events (pain, SSEs, etc.) have been extremely important to patients with solid tumors [16], most studies conducted in cancer have found patients value OS or progression-free survival as the most important attribute when considering treatment options [17, 24–26]. It is possible that the differences between our findings and those of the literature could be a function of tumor type (CRPC vs. other tumors), geography (Japan vs. the West), or another aspect of the methods (e.g., a more restricted range of OS levels vs. a wider range of OS levels; older, late-stage patients vs. younger, earlier-stage patients). However, the results do suggest that patients with CRPC in Japan place considerable value on their symptom experience when expressing a treatment preference.

Of course, OS was still viewed as an important attribute. Indeed, it was considered 30–40 % more important than the method of administration and time to an SSE. Consistent with many studies conducted in cancer [16, 17, 25, 26], method of administration held only a modest association with medication choice. Oral administration was most positively associated with choice, while a 1-h IV infusion every 3 weeks was most negatively associated with choice. Interestingly, patients had a slight preference for “some radiation” over “minor radiation,” even though “some radiation” would require extra effort on the part of the patient to ensure his bodily fluids do not contaminate others. It is unclear why this might be. It is possible the most salient aspect of these administration options is the injection itself (rather than the accompanying radiation). If so, the differences between “some radiation” and “minor radiation” may be merely due to chance. Additional research would be necessary to examine this issue.

A plateau effect was observed with respect to time to an SSE. Particularly, there seemed to be little benefit to extending time to an SSE from 14 to 16 months. In contrast, there was a considerable benefit to extending time to an SSE from 10 to 14 months.

Work impairment was most weakly associated with medication choice. In part, this may be due to the fact less than 25 % of patients held some form of employment. The effect of 3 lost days and 5 lost days on preference was similar; having to miss work at all mattered more to patients than the number of days missed.

Although difficult to draw firm conclusions given the small sample size, differences in preferences were examined as a function of demographic and health characteristics. Few differences were observed, suggesting the potential homogeneity of treatment preferences among those with CRPC in Japan. Of primary interest was the comparison between symptomatic and asymptomatic patients. Symptomatic patients placed significantly more importance on delaying time to an SSE, suggesting that patients’ actual experience with an SSE makes them appreciate the value in delaying these events, which may not be the case for patients who have not experienced an SSE. No other differences in preferences were observed between symptomatic and asymptomatic patients.

These study results help to provide insight into the patient experience with CRPC treatments in Japan. A recent American Society of Clinical Oncology statement paper reinforced the importance of capturing the patient perspective in defining the value of a treatment option in oncology [27]. Because preferences can be unique to each person, it is important to present the various clinical benefits and risks to ensure patients are kept informed throughout the treatment decision-making process. Our results help to identify clear trends in the treatment attributes that matter most to CRPC patients, which can facilitate discussions between physicians and their patients to select the best treatment course.

### Limitations

A few limitations should be noted. Due to sample selection during recruitment, we were only able to analyze data from patients who had already been pre-screened by their physicians, and the total number of patients who were initially screened to obtain our final sample could not be verified. Hence, patients who were healthy enough to participate and were interested in research may be over-represented in our sample, thereby raising the possibility of selection bias. While we could not confirm the extent to which the current study’s sample represented the broader Japanese population of CRPC patients, we made sure to recruit patients from institutions in both east and west Japan, and the clinical and demographic features of our sample (e.g., age, ECOG, BMI) were generally in line with those reported in prior studies [28, 29]. Although efforts were made to ensure a representative sample, we could not control the precise ratio of subgroups that may appear in the CRPC population. Therefore, the results may not generalize to the entire CRPC population.

The data collected in the DCE were based on responses to hypothetical choice profiles. These choices were intended to simulate possible clinical decisions, but obviously do not have the same clinical, financial or emotional consequences of actual decisions. Although we identified the key clinical features that differ across PC treatment options, there are a multitude of other factors (e.g., presentation of treatment options by the physician, Internet research, family/friend opinion, etc.) that would be present in a real-world choice selection that could not reasonably be accounted for in our controlled experiment. As a result, when confronted with a real choice, preferences may differ from our DCE results. Thus, differences can arise between stated and actual choices. Finally, duration of treatment and patient burden may also have affected patient preferences, although subgroup analysis using patient clinical background did not show any significant differences in preference.

## Conclusions

CRPC patients were more concerned about reduced quality of life derived from side effects of treatment than extension of survival, which may impact shared decision-making between patients and physicians. In particular, CRPC patients in Japan place particular importance on the risk of fatigue, reducing the risk of bone pain, and OS when expressing a preference for a hypothetical PC treatment. Treatment preference largely did not vary as a function of symptomatic status except in the case of time to an SSE. Patients who were asymptomatic placed significantly more importance on delaying an SSE when expressing medication preferences.

## Abbreviations

BMI: Body mass index
CCI: Charlson comorbidity index
CI: Confidence interval
CRPC: Castration-resistant prostate cancer
DCE: Discrete-choice experiment
ECOG: Eastern Cooperative Oncology Group
IV: Intravenous
mCRPC: Metastatic castration-resistant prostate cancer
OS: Overall survival
PC: Prostate cancer
RI: Relative importance
SD: Standard deviation
SSE: Symptomatic skeletal event
