# Erratum to: Patient preferences for treatment of castration-resistant prostate cancer in Japan: a discrete-choice experiment

**DOI:** 10.1186/s12894-017-0210-x

**Published:** 2017-03-28

**Authors:** Hiroji Uemura, Nobuaki Matsubara, Go Kimura, Akito Yamaguchi, Dianne Athene Ledesma, Marco DiBonaventura, Ateesha F. Mohamed, Enrique Basurto, Ian McKinnon, Ed Wang, Kristen Concialdi, Aya Narimatsu, Yasuko Aitoku

**Affiliations:** 1 Department of Urology and Renal Transplantation, Yokohama City University Medical Center, Yokohama, Japan; 2 Division of Breast and Medical Oncology, National Cancer Center Hospital East, Chiba, Japan; 3 Department of Urology, Nippon Medical School, Tokyo, Japan; 4 Division of Urology, Harasanshin General Hospital, Fukuoka, Japan; 5 Bayer Yakuhin, Ltd., 2-4-9, Umeda, Kita-ku, Osaka, 530-0001 Japan; 6 Kantar Health, New York, NY USA; 7 Bayer Healthcare, Whippany, NJ USA

## Erratum

After publication of this work [1] it was noticed that there was a typographical error in the Conclusions. The letter ‘a’ in ‘asymtoptomatic’ should have been removed in the concluding statement as it is inconsistent with the Results and Discussion. The statement in the Conclusions should read: ‘Patients who were symptomatic placed significantly more importance on delaying an SSE when expressing medication preferences.’

The original article was corrected. The publisher apologises for this error.
